# Contribution of Sex-linked Biology and Gender Roles to Disparities with Trachoma^1^

**DOI:** 10.3201/eid1011.040353

**Published:** 2004-11

**Authors:** Paul Courtright, Sheila K. West

**Affiliations:** * Tumaini University, Moshi, Tanzania;; † Johns Hopkins University, Baltimore, Maryland, USA

**Keywords:** Gender, trachoma, trichiasis, blindness, conference report

## Abstract

Globally, trachoma is the leading infectious cause of blindness. Survey data consistently show that trachoma-related blindness is two to four times higher in women than men. Tracing the increased risk for trachoma and its consequences for women suggests that other factors besides biology may contribute. Understanding the reasons for the excess risk for and consequences of trachoma in girls and women requires examining a number of issues: Are girls and women more biologically susceptible to the consequences of infection with *Chlamydia trachomatis*? Could other factors help explain the excess of conjunctival scarring and trichiasis in women? Do gender roles affect the risk for trachoma and its consequences? Are women more likely to have recurrence after trichiasis surgery compared to men? This article explores the answers to these questions.

## Abstract 2

Gender roles, rather than biology, dictate why trachoma is more common in women.

Blindness is a major public health concern globally: approximately 50 million people are blind, and three times that number are visually impaired. Unless marked improvements are made in numbers of healthcare personnel, infrastructure, and use of services, 75 million cases of blindness will likely occur by 2020.

Recent evidence has shown that women account for approximately 64% of global blindness; an age-adjusted prevalence 39% higher than in men (*1*). Cataract, the leading cause of blindness in the world, occurs more commonly in women than men (*2**–**3*). Paradoxically, in developing countries the use of cataract surgical services among women is considerably lower than among men (*4*). Other leading causes of blindness include glaucoma, diabetic retinopathy, and trachoma.

Trachoma is often referred to as the leading cause of preventable blindness in the world. It is the leading infectious cause of blindness, followed by diseases such as onchocerciasis and measles. Systemic diseases such as leprosy and HIV/AIDS also lead to blindness, although much less frequently.

Trachoma is a chronic infectious eye disease affecting marginalized population groups throughout many countries of Africa, the Middle East, Asia, and a few settings in Latin America. *Chlamydia trachomatis*, the infective agent, has no known animal reservoir. The manifestations of trachoma vary depending upon the number of episodes of infection, severity, and the persistence of infection. Trachoma generally occurs early in life through physical transmission of *C. trachomatis* to the eye by hands, flies, or cloth. The pool of chlamydiae in the community generally resides in preschool-age children (*5*), and transmission is easily facilitated by poor hygiene, scarcity of water, and crowded living conditions. The highest prevalence of active trachoma in hyperendemic areas is found among children 1–3 years of age. Adult women are also more likely to have evidence of active disease and infection.

A single episode of infection will not lead to sequelae; however, repeated bouts of infection and active disease as well as persistent infection will lead to scarring of the upper tarsal conjunctiva. Approximately 10% of children have persistent infection and many are actively and consistently shedding infectious agent (*6*). The severity of active disease will generally dictate the severity of scarring. Among those with tarsal scarring, a small proportion will have thickening of the tarsus and deformation of the lid, whereby the malposition of eyelashes leads to abrasion of the cornea and, in some cases, corneal ulceration and scarring. Consequently, trachoma occurs throughout patients' lives, exhibiting different signs and symptoms at different stages; Figure 1 gives an example of the changes in disease and its sequelae in trachoma-endemic countries.

**Figure 1:**
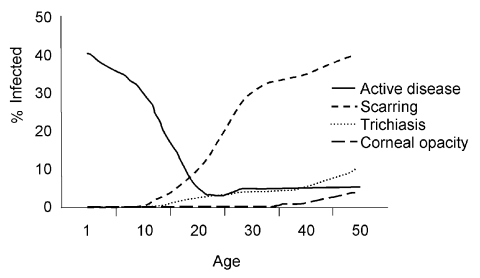
Trachoma as a disease that occurs throughout the life of a person.

Estimates of the number of people affected and blinded by trachoma have been recently revised; approximately 80–85 million people now have active trachoma, approximately 8 million have trichiasis, and 3 million are blind. Most of those affected are found in sub-Saharan Africa, the Middle East crescent, and parts of Asia. The distribution of trachoma corresponds with that of poverty in much of Africa and Asia. Conditions that facilitate the transmission of *C. trachomatis*, such as household crowding and poor access to and use of water, define risk (*7*). Because of changes in disease endemicity, active disease and trachomatous trichiasis are less correlated than they were 50 years ago; for example, active trachoma has disappeared in most areas of China, where it was once endemic. There are, however, approximately 2.5 million people in China blind because of trachoma (*8*). Trichiasis also remains common in countries such as Vietnam, where active disease rates are relatively low. Trachoma has been eliminated as a public health problem in these settings and others (such as Tunisia, Saudi Arabia, and Oman) through improved socioeconomic status, improved environmental and public health infrastructures (water supply, latrines), and changed behavior. Nevertheless, because infections accumulated in the past, trichiasis is likely to develop in many persons in these countries.

Productivity losses attributable to trachoma are conservatively estimated at U.S.$2.9 billion annually. This figure is likely an underestimate of the social cost of the disease (*8*).

## Trachoma as a Gender-associated Health Issue

Survey data from virtually every trachoma-endemic setting have consistently shown that trachoma-related blindness is two to four times higher in women compared to men (*9**–**12*). Survey data also show that women have an excess risk for corneal opacity, trichiasis, and scarring; women account for 60% to 85% of all cases of trichiasis in the community (*11**–**14*). Tracing the excess risk for and complications of trachoma borne by women through the course of disease suggests that factors other than biology are involved. While evidence shows that, by the age of 40, women are more likely to have severe conjunctival scarring compared to men, the relative effect is not as high as expected, if a linear association is assumed. The further back along the course of the disease in a patient, evidence becomes less and less clear. In young children, the focus of most active trachoma in a community, the prevalence of active disease seems to vary, with just a slight excess risk for girls. In some surveys, such as recently in Dalocha District in Ethiopia, while 11 of 18 trichiasis cases were in women, in children <10 years of age, 21.1% of boys and 13.5% of girls had active trachoma (*13*). However, in another district in Ethiopia, girls and women were 1.63 times more likely to have active trachoma compared to boys and men (*11*). Preschool girls in Tanzania had slightly higher rates of active trachoma than boys; including adults in the findings showed that female patients had a twofold excess risk for active trachoma compared to male patients (*14*). In most settings, the differences in prevalence of active trachoma between girls and boys are not as striking as they are in women and men (*12**,**15*).

## Excessive Effect of Trachoma on Women

Understanding the reasons for the excess risk for and complications of trachoma in girls and women requires examining a number of issues. Are females more biologically susceptible to the consequences of infection with *C. trachomatis*? Could other factors (e.g., number of episodes, persistence of disease, higher bacterial loads) help explain the excess conjunctival scarring and trichiasis in women? Do gender roles affect the risk for trachoma and its consequences? Are women more likely to have recurrence after trichiasis surgery compared to men?

## Biologic Susceptibility to Consequences of Infection with *C. trachomatis*

Research in developing countries has not shown that women or girls are more biologically susceptible to the consequences of infection with *C. trachomatis*. However, research in industrialized countries has demonstrated a higher prevalence of dry eye syndrome in women, likely due to hormonal factors. If these findings are applied to trachoma-endemic settings, they would suggest that corneal damage is more likely to develop in women with trichiasis than men. The paucity of longitudinal data analyzed by sex limits our ability to confirm this hypothesis.

## Other Factors Explaining Excess Conjunctival Scarring and Trichiasis in Women

Recent research in Tanzania suggests that, while girls have only slightly higher prevalence of active disease than boys, they account for most of the community load of the community load of *C. trachomatis* infection (A. Solomon, pers. comm.). In a different population in Tanzania, when the group of children with a heavy bacterial load were compared to noninfected persons, those with a heavy bacterial load were more likely to be younger and to be female (odds ratio 1.64, 95% confidence intervals 1.1–2.5) and to live in a house with at least one other person with heavy infection (*16*). In a separate study in the same district, antimicrobial treatment was provided to all residents. At the 2-month follow-up, the predictors of infection were being younger and female and having trachomatous inflammation at baseline assessment before treatment. At 6 months, the strongest predictor of infection was infection at 2 months; 95% of those with a severe infection at 2 months were infected at 6 months (*17*). Despite high rates of antimicrobial coverage in this community, *C. trachomatis* was reemerging, even among those treated.

These studies suggest that infection loads are higher in girls and women and that persistent infection is more common in girls. Longitudinally, this hypothesis is supported by the 7-year follow-up results on conjunctival scarring. Of girls with severe active trachoma at the beginning, 33% eventually had scarring compared to 22% of boys with similarly severe active trachoma (*18*).

Few studies have measured infection with *C. trachomatis* in adults. In a study of women, infection in adulthood, although not common, increased the risk for trichiasis 2.5-fold (*19*). The few studies with information on infection show that adult women are more likely than adult men to be infected (*5**,**20*). These data justify treating adults, particularly women.

In a follow-up study of women in Tanzania, evidence for persistent infection existed, as several women were infected at follow-up with the same genovar (*21*). Whether men have more or less susceptibility to persistent infection is not known.

## Effect of Gender Role on Risk Factors for Infection

Women and girls are the primary caregivers in most societies in developing countries. Proximity to children exposes women to repeated infection more than men and is likely a primary reason for the greater effect of active disease (*22*). The close association of mothers with children makes them more likely to continue to acquire infection and exposes them to the risk for persistent infection. Ongoing infection and scarring may explain the almost four times greater risk compared to men of developing trichiasis. In central Tanzania, women with preschool-age children appeared more likely to have active trachoma than similarly aged women without preschool-age children. Young girls have the role of childcare in this setting, and active disease is associated with the status of mother or caretaker (*14*). However, a case-control study of trichiasis in women did not find a significant difference between childcare activities in those with trichiasis compared to those without trichiasis; the study design did not permit an evaluation of infection in the children themselves, which is the key factor (*23*). That study did find an increased risk in women whose mothers also reported trichiasis, which suggests a possible genetic or shared environmental component to trichiasis. The absence of rigorous behavioral research has limited our ability to understand how gender roles contribute to the progression from infection to clinical disease and its sequelae.

In many Islamic countries the use of kohl and other traditional products for preventing eye diseases, treating eye diseases, and "brightening" the eyes is common and can be implicated in disease transmission within the household and community (*24*). In addition, some of these traditional products are applied by everting the lid, moistening the product with saliva, and rubbing the compound directly onto the tarsus. This procedure may further scar the tarsus and spread bacteria from the nasopharynx. These products are used among both young boys and girls, but continued use in adulthood is limited primarily to women. This practice may be another way in which gender roles contribute to the excessive effects of trachoma in women.

## Recurrence after Trichiasis Surgery

Recurrence after surgery to correct trichiasis is not uncommon. Recurrence rates reflect both poor quality of surgery (recurrence usually occurring within 6 months of surgery) or other pathologic processes (e.g., continued contracture of the tarsal conjunctiva, reinfection). Recurrence, measured cross-sectionally, may reflect both of these conditions; measured prospectively, determining the apparent contribution of each factor is easier. Most studies of the outcome of trichiasis surgery had few patients enrolled and lacked adequate power to compare differences between men and women (*25*). Omani women and men, with 3- to 5-year follow-up data, had unequal rates of recurrence. Overall, 61.3% of surgeries in women were associated with recurrences compared to 48.9% among men (*26*). West et al., in their large series of cases in Tanzania, found no difference by sex in recurrence rates up to 8 years (E. West, pers. comm.). Cross-sectional studies indicate that women are more likely to have recurrence compared to men. In the recent Egyptian study, trichiasis recurred after surgery in 44.4% of women compared to 37.7% of men (*10*).

**Figure 2:**
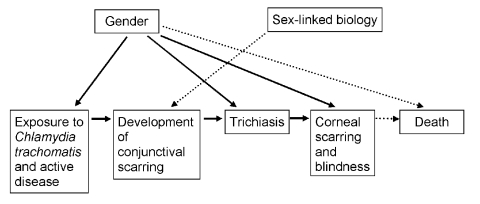
Contribution of gender and sex-linked biology to the progression to blindness in trachoma.

Possible reasons for the differences are multiple and may overlap: Women may have had more severe trichiasis before surgery, which is a known risk factor for recurrence (*27*). In some settings, women use surgical services less or may wait until trichiasis is more advanced before surgery. Finally, reinfection may play a role in recurrence. As noted, infection in adulthood increases the risk for trichiasis 2.5-fold (*19*). In the Omani study (*26*), recurrence was approximately three times higher in persons with infective conjunctivitis compared to those without conjunctivitis. All these potential explanations point to gender role–mediated factors more than sex-linked factors. Gender roles in most societies in trachoma-endemic countries account for the variation in prevalence of infection and sequelae of infection seen in men and women (Figure 2).

## Public Health Approaches to Trachoma Control and Gender Equity

Eliminating trachoma as a blinding disease is the goal of the Alliance for the Global Elimination of Trachoma by 2020. The Alliance has adopted a four-part strategy, referred to as the SAFE strategy, which includes surgery to correct trichiasis, antimicrobial agents to treat active trachoma, and face washing and environmental changes to prevent transmission. Adopting the SAFE strategy, while not explicitly gender-sensitive, would necessitate considering gender in implementation.

Few national trachoma control programs monitor use of trichiasis surgery separately for men and women. Fewer still have adequate information on the proportion of trichiasis patients who have had surgery. This lack of this information limits evaluation of program progress, gender based or not. Data from the Tanzania and Vietnam programs suggest that these countries have gender equity in trichiasis surgery (*28*). However, programs directed at increasing the number of persons receiving surgery to correct trichiasis have generally not been explicitly gender-sensitive, and publications on uptake of services have rarely compared men and women. Gender equity in use thus remains unknown in most settings.

Similarly, trachoma control programs have tended to measure antimicrobial coverage as a single, communitywide or districtwide measure, without assessing rates of compliance by age and sex. Research on compliance from Rombo district in Tanzania showed that compliance with azithromycin treatment was higher among women (80.2%) than men (73.3%). However, as pregnant women are not eligible to receive azithromycin and must use topical tetracycline, interpreting coverage findings is not straightforward (*29*). The authors suggested that, since childcare is primarily the concern of women, they were more likely to attend the distribution of azithromycin by bringing their children and thus are more likely than men to receive drugs for themselves. Furthermore, the authors suggested that patient or recipient expectations contributed to compliance and that the expectations of women were influenced to a greater extent than those of men by their children's illness (N. Desmond, pers. comm.). Current recommendations promote a minimum antimicrobial coverage (whole population) of 80%; evaluation is needed to assess if women are more or less likely to get an antimicrobial agent.

Although the antimicrobial agent is currently donated free-of-charge, the cost of distribution remains high (approximately U.S$0.50 per dose) (*30*). This cost is borne by various governmental and nongovernmental agencies; however, how long this can continue is not clear. Willingness to pay for research in Tanzania has demonstrated that those at higher risk for trachoma were less willing to pay for future treatment. This group included female-headed households (*31*). Only higher maternal education was associated with willingness to pay.

Approaches to preventing trachoma in the SAFE strategy, namely, face washing and environmental changes, have not been researched to the same extent that surgery and antimicrobial use have. Research in the early 1990s demonstrated the effectiveness of face washing in trachoma reduction (*32*). Cross-sectional studies of risk factors have consistently showed a strong association between the presence of active trachoma and the absence of good sanitary conditions (primarily the absence of latrines and the high concentration of flies). Assessment of environmental conditions (including water supply) is household- or community-based, rather than individually based, thus limiting the ability to evaluate gender-specific characteristics. Nevertheless, the fact that women and girls are primarily responsible for water collection, face washing, and cleaning (if done) of latrines suggests that introducing improved infrastructures will have the greatest effect on women, both in terms of eliminating trachoma and improving quality of life. Women's and girls' roles also suggest that most hygiene and environmental components of trachoma control will fall to women as well. Thus, for example, in the absence of water in the villages, convincing women to use scarce water that they must collect for washing purposes is difficult.

Trachoma remains a major problem, particularly among girls and women, in much of sub-Saharan Africa, areas of the Middle East crescent, and pockets of Asia and South America. Eliminating trachoma, while possible, will require a rededication to prevention strategies and a focus on disease control as a gender-sensitive intervention.
